# Dissolving Polymer Microneedles for Transdermal Delivery of Insulin

**DOI:** 10.3389/fphar.2021.719905

**Published:** 2021-09-22

**Authors:** Na Zhang, Xinxin Zhou, Libo Liu, Lini Zhao, Hui Xie, Zhihang Yang

**Affiliations:** ^1^ Department of Electrical Diagnosis, Central Hospital Affiliated to Shenyang Medical College, Shenyang, China; ^2^ Liaoning Provincial Key Laboratory of Behavior and Cognitive Neuroscience, Shenyang Medical College, Shenyang, China; ^3^ Department of Liaoning TCM Academy, Liaoning University of Traditional Chinese Medicine, Shenyang, China; ^4^ Department of Neurobiology, School of Life Sciences, China Medical University, Shenyang, China; ^5^ Department of Pharmacology, Shenyang Medical College, Shenyang, China; ^6^ Department of Histology and Embryology, College of Basic Medicine, Shenyang Medical College, Shenyang, China; ^7^ Department of Physiology, College of Basic Medicine, Shenyang Medical College, Shenyang, China

**Keywords:** dissolving microneedles, insulin, transdermal drug delivery, diabetes treatment, PVA

## Abstract

It’s of great significance to develop insulin-loaded dissolving microneedles (MNs) which are fabricated with various methods and materials for transdermal delivery of insulin to effectively and efficiently treat diabetes. In this work, we present a kind of FITC-insulin tip-loaded dissolving MNs fabricated with the mixture of polyvinyl alcohol (PVA) and sucrose using homemade PDMS MNs mold under vacuum conditions. The uniform appearance of MN arrays contributes to controlling the drug dosage well as required. Sufficient mechanical strength for penetrating tough stratum corneum can be obtained by vacuum frozen-drying for at least 6 h after peeling MNs off the mold. About 90% of the FITC-insulin is localized in the conical MN tips and can be released into the skin within 2 min after insertion. The *in vivo* insulin absorption study and hypoglycemic effect in diabetic mice demonstrate that the proposed insulin-loaded MNs can efficiently deliver the insulin to the systemic circulation and exhibit a similar effect to hypodermic injection on hypoglycemic administration. Together these results suggested that the efficient MN fabrication process proposed in this work shows great potential for mass production and practical application of drug-loaded dissolving MNs in the future.

## Introduction

Currently, the treatment for insulin-dependent diabetes mellitus patients mainly involves obtaining a normal blood glucose value (below 8.0 mmol/L prior to large meals for adult diabetes patients) by delivery of exogenous insulin *via* conventional injection pen or insulin pump multiple times per day (Rodger, 1991). However, frequent injections using traditional hypodermic metal needles may cause physical pain, wound infection, tissue necrosis and nerve damage, which is inconvenient and leads to poor patient compliance (Chu, 2005). To address these issues, many alternative methods of insulin delivery have been investigated, including intranasal, pulmonary, oral and transdermal administration (McAllister et al., 2003; Prausnitz, 2004; Lassmann-Vague and Raccah, 2006; Chen et al., 2011; Chaturvedi et al., 2013; Mo et al., 2014). Microneedles, as third-generation drug delivery systems targeting their effects to break through skin’s barrier layer of stratum corneum in a minimally invasive manner, are currently progressing through clinical trials for transdermal delivery of macromolecules, such as insulin, parathyroid hormone and rabies vaccination (Prausnitz and Langer, 2008; Fukushima et al., 2010; Kim et al., 2012; Arya et al., 2016).

Recent studies indicate that transdermal insulin delivery into diabetic systemic circulation *via* dissolving microneedles (MNs) takes considerable effect on reducing blood glucose level and even exhibit the same pharmaceutical efficacy as conventional subcutaneous (SC) injection of insulin solution (Ito et al., 2006; Ito et al., 2012a; Liu et al., 2012; Ling and Chen, 2013; Chen et al., 2015). At the same time, novel dissolving MNs seems to be pain-free, bio-safe, patient-friendly and self-applicable compared with conventional hypodermic needles in the application (Sullivan et al., 2008). Dissolving MNs are not medical devices for drug delivery, such as hollow or coated MNs (Gill and Prausnitz, 2007; Gupta et al., 2009), but pharmaceuticals that dissolve in the skin tissue and release encapsulated drug subsequently (Ito et al., 2012a). Matrix of dissolving MNs mainly consists of biodegradable and biocompatible materials including gelatin and starch, polysaccharide, chondroitin sulfate, sodium carboxymethyl cellulose, Gantrez R AN-139, dextran, hyaluronic acid and polyvinyl alcohol (PVA) (Chen et al., 2015). As the reported fabrication methods, dissolving MNs can be prepared through droplet-born air blowing (Kim et al., 2013), MN molds casted under centrifugation or vacuum condition (Yang et al., 2012). Except for the matrix material and preparation process, the geometry and mechanical structure of dissolving MNs also have a significant influence on the MN performance in the process of application.

When inserting drug-loaded dissolving MNs into the skin, needles with conical or pyramidal shapes could not be inserted completely into the subcutaneous tissue from top to bottom due to the elastic skin deformation (Zhu et al., 2016). Only part of the drug localized in the MN tip can be absorbed into the interstitial fluid effectively, which reduces the efficiency of transdermal drug delivery *via* dissolving MN and also decreases the accuracy of drug dose delivered into the body as expected (Ito et al., 2012b; Wang et al., 2015; Yang et al., 2015). As we know, precise insulin dosage is very important for diabetic patients. Generally, an overdose would result in serious hypoglycemia symptoms and a shortage of insulin would not reach the expected therapeutic effect to maintain normal blood glucose levels. Many attempts had been made to control the drug dosage precisely and improve the drug delivery efficiency by designing novel MNs, such as tip-loaded coated MNs (Kim et al., 2016; Liu et al., 2016), bubble MNs (Chu et al., 2010) and two-layered dissolving MNs (Ito et al., 2012a).

In this study, we reported a novel fabrication process of FITC-insulin tip-loaded dissolving MNs. PVA was applied as the matrix material of the dissolving MNs not just for biocompatibility reasons, but also because of its excellent mechanical properties and processability. Moreover, considering the stability of insulin encapsulated within the microneedles, sucrose was added to the MN formulations as stabilizers in this work. Homemade shape-controllable polydimethylsiloxane (PDMS) mold carved with laser engraving technology was used to prepare MN patches. The concentration and volume of FITC-insulin solution can be controlled well when filling a certain dose of drug into the MN cavities as required. Additionally, PDMS with porous structure is air-permeable but water-impermeable and the breathable property is suitable for casting drug solution or viscous polymer gel into the micro-cavities by applying a vacuum at the opposite side of the mold. This mild molding process avoids lots of disadvantages such as organic solvents, heat or UV light irradiation, which protects the bioactivity of insulin from damage and shows obvious potential to reduce pollution and energy cost largely if mass production of drug-loaded dissolving MNs becomes realized someday. To assess the availability of dissolving FITC-insulin loaded MNs, we prepared MNs with different insulin dosages and tested their mechanical properties. *In vitro* MN dissolution kinetics and drug delivery efficiency were also investigated. Additionally, we created streptozotocin-induced diabetic models using Balb/c mice (Yin et al., 2006) and determined the hypoglycemic effect *in vivo* after transdermally inserting FITC-insulin loaded MNs and then injecting insulin solution into diabetic mice as compared simultaneously.

## Materials and Methods

### Materials

Polyvinyl alcohol (78% hydrolyzed, *M*_w_ = 6,000 Da) and D (+)-Sucrose (≥99% Ultra-Pure Grade, *M*_w_ = 342.29 Da) were purchased from Acros Organics (New Jersey, United States). FITC-Insulin was provided by ZhongKeChenYu Biotech Co. Ltd (Beijing, China). Streptozotocin was provided by AbMole Bioscience (Shanghai, China). Fluorescence microscopy (Olympus SZX7) was obtained from Micro-Shot Technology Co., Ltd (Guangzhou, China). Model ESM301 Force Test Stand and Series 5 Digital Force Gauge were purchased from Mark-10 Corporation (Copiague, United States). *In Vivo* Imaging Systems (IVIS Spectrum) was purchased from Caliper Life Sciences (Massachusetts, United States). All chemicals were used as received without additional treatment.

### Preparation of PVA/Sucrose MN Matrix Gel

The formula of PVA and sucrose mixed gel solution has a great influence on the mechanical strength of MN support structure and the profile of drug distribution in the whole needles. The polymer solution used to fill the MN mold cavities was prepared as follows, dissolving PVA powder in deionized (DI) water by stirring with a magnetic stirrer continuously at a rotational speed of 200 r/min for 4 h at 90°C in a sealed glass container, then added sucrose particles into PVA solution at a certain mass ratio (PVA/sucrose/DI water, 8: 6: 15) and stirred for another 30 min under the same condition. After that, the mixture solution was placed at room temperature for 1 h to cool down and subsequently put into the vacuum oven to remove the encapsulated tiny air bubbles to acquire homogeneous and transparent gel solution. The viscosity of PVA/sucrose polymer solution was measured to be 3,700 mPa s with a rotary viscosity meter (NDJ-1) at 25°C. The obtained PVA/sucrose hydrogel is highly hydrophilic and biocompatible. Meanwhile, it’s mechanically strong and slightly elastic at dry and hydrated states respectively, which contributes to filling MN mold cavities and demolding MN patches from the mold.

### Fabrication of FITC-Insulin Loaded Microneedles

**FIGURE 1:**
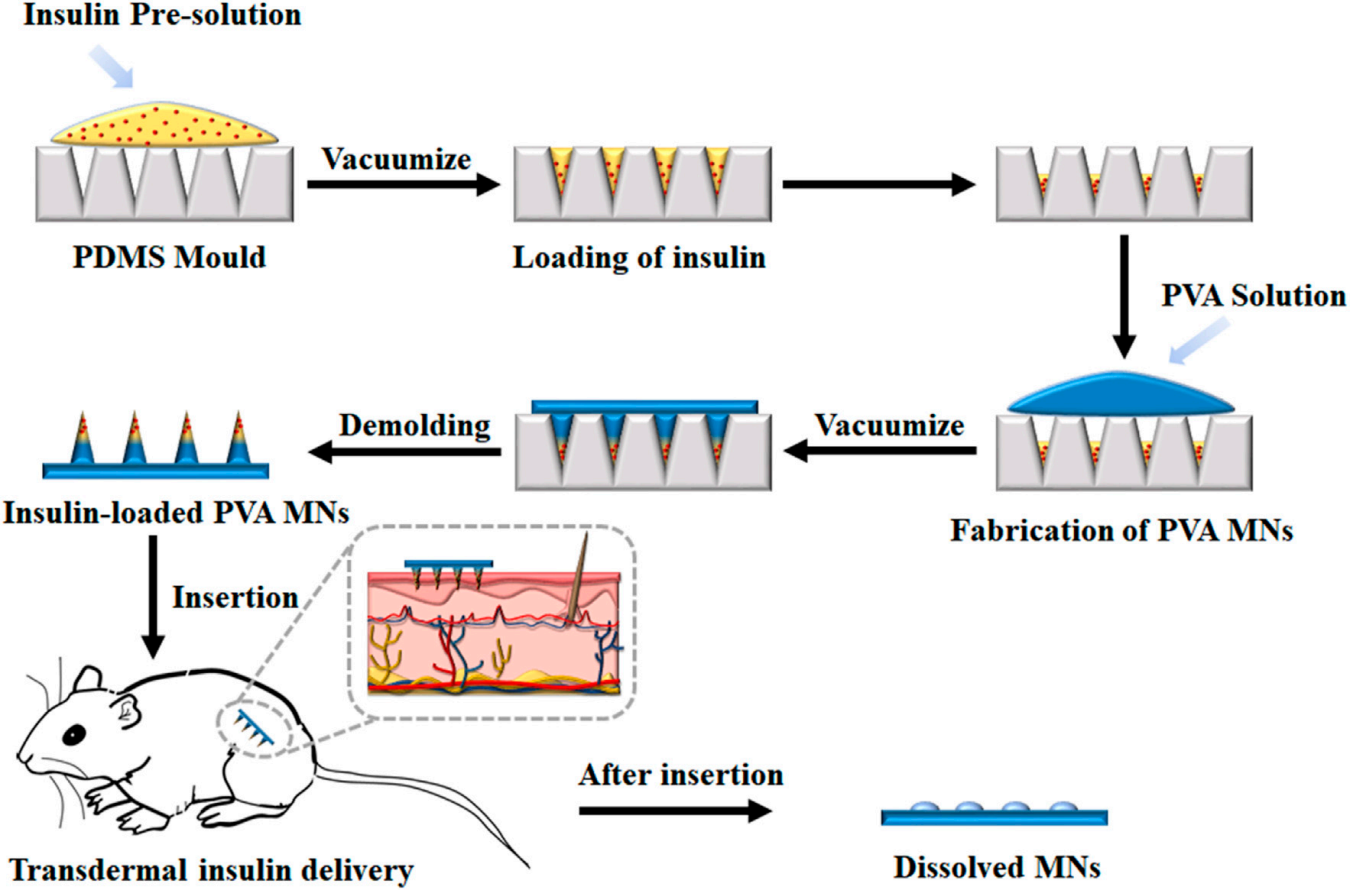
Schematic illustrations of insulin-loaded MNs patch fabrication process and its application.

The porous structure of PDMS allows air permeating freely but resists liquid passing through. The array size of MN mold cavities is 10 × 10, with 750 µm depth, 300 µm base diameter of each conical cavity, and 500 µm tip-to-tip spacing. The volume of each needle is about 2 µl. A cast processing was used to prepare the drug-loaded dissolving MNs (Figure 1). Briefly, about 100 µl of FITC-insulin solution with a certain concentration of 1 mg/ml was casted onto the dust-free PDMS mold surface and sealed the MN mold cavities tightly, and MN fabrication with other concentrations of 2, 3, and 4 mg/ml was completed respectively in separate experiments. Then the stable vacuum condition (−0.1 MPa) was applied at the opposite backside of the casted mold surface. FITC-insulin solution was inhaled slowly and filled the cavities fully within 15 min, and then redundant drug solution on the mold surface was removed by pipetting and recycled for later use. After evaporating the drug solution filled in the mold cavities at room temperature for 20 min, the PVA/sucrose gel with about 1 mm thick was coated onto the mold surface and filled the cavities for another 1 h at the same vacuum condition as previous. Then the whole system was dehumidified as follows, the samples were frozen at −40°C for 1 h, and then vacuumed at -101 kPa at −40°C for 12 h and 0°C for 1 h in the freeze dryer (Boyikang, VFD-1000, Beijing). Then the MN arrays were detached from the mold cavities using adhesive pads and observed using a fluorescence microscopy. All the prepared MN patches used for animal test were sterilized with ultraviolet (UV) lamp.

### Test of Mechanical Strength and Insertion Ratio of MN Patches

To evaluate the mechanical property of MN arrays drying for a different time after demolding, FITC-insulin loaded MN patches just peeling off were dried in the freeze dryer at 0°C and −101 kPa for 2, 4, 6, 8 and 10 h respectively. The mechanical property was measured using a displacement-force test machine (Mark-10, Force Gauge Model, United States). In detail, the MN patch was put on the flat stainless steel platform with needle tips upward and the original array size was cut into 3 × 3, leaving nine needles in the patch center. A pressure sensor with a horizontal plate probe in the head applied a perpendicular axial force to press the needle tips at a constant traveling speed of 0.5 mm/min. Then the reacting force that needle tips applied conversely to the probe plate was subsequently recorded as the pressure displacement increasing constantly, and test finished until the measured force climbed to the maximum setting value of 10 N (Figure 3).

Additionally, to evaluate the different insertion capabilities of drug-loaded MNs drying for a different time, insertion ratio was determined by piercing MN arrays into fresh hairless porcine cadaver skin with a homemade MN applicator quickly. Then wipe off the residual FITC-insulin on the skin surface with an alcohol pad and observing the penetrated needle holes with a fluorescence microscope. The insertion ratio for each MN array was calculated by dividing the number of fluorescent spots on the skin by the number of total array needles.

### *In Vitro* Test for MN Dissolution Kinetics and Drug Delivery Efficiency

To evaluate the *in vitro* dissolution profile of FITC-insulin loaded MNs, prepared MN patches were inserted into fresh hairless porcine cadaver skin (Pel-Freez, Roger, AR) using a homemade applicator. Hairless skin was prepared by removing the hair on the skin surface using a stainless razor before insertion. The different groups of MN patches were manually pressed into the skin and peeled off after being embedded subcutaneously for 5, 10, and 30 s, 1, 2, 5, and 10 min respectively. Then alcohol pads were used to clean and collect the residual drug on the skin surface. Both bright field and fluorescence images of the MN arrays and porcine skin containing FITC-insulin were taken using a fluorescence microscopy.

To determine the drug delivery efficiency of FITC-insulin loaded MNs, the prepared MN patches containing different doses of FITC-insulin were administered to the porcine cadaver skin. The drug delivery efficiency was determined by measuring the initial drug content in the MNs and the residual drug content in the MNs and on the skin surface after penetration. To determine the FITC-insulin standard curve, FITC-insulin standard solutions of different concentrations were prepared and measured their absorbance. Alcohol pads were used to collect residual drug on the skin surface and then were soaked in 1 ml PBS solution for later determination. The intact MN patches and residual MN substrate were dissolved as above (n = 5 for each group). All the concentration of FITC-insulin solution was quantified using a fluorescence microplate reader (Thermo Fisher Science OY, Vantaa, Finland).

### *In vivo* Application of Insulin-Loaded Microneedles in Diabetic Mice

The Shenyang Medical College Institutional Animal Care and Use Committee approved all animal care and experimental protocols used in the studies. Female Balb/c mice (∼15–20 g) obtained from the Institute of Laboratory Animal Sciences were acclimatizing to standard laboratory conditions for more than 2 weeks before any experiments.

To evaluate the hypoglycemic effect of FITC-insulin loaded MNs, typeⅠ diabetic models were created according to the published work (Yin et al., 2006). Balb/c female mice weighing 16–18 g were fasted for 6–8 h before being anesthetized in the induction box with flowing gas mixed by isoflurane and oxygen (1:2 volume ratios) at the gas velocity of 20 ml/min, and then STZ with a dose of 200 mg/kg was injected into healthy mice by intraperitoneal injection. To avoid STZ hydrolyzing slowly, dry STZ powder should be dissolved in sodium citrate buffer (pH 4.5) in 5 min just before injection, and obtained STZ solution with a concentration of 10 mg/ml at room temperature. Generally, diabetic mice models were successfully created when blood glucose (BG) level reached 300–550 mg/dl after STZ onset of effect within 2 days.

Prior to determining the hyperglycemic effect of FITC-insulin MN patches, diabetic mice were fasted for 12 h, while water was fed ad libitum. Then animals were anesthetized as previously. The abdominal and back regions were shaved using an electrical shearing knife and depilated with depilatory cream carefully. After that, the initial value of BG was recorded. Different groups of diabetic mice were administrated as follows: 1) MNs insertion group, where 0.05, 0.1 and 0.2 U of FITC-insulin loaded MNs were applied onto hairless abdominal skin and removed after being pressed with a homemade applicator for 2 min; 2) Subcutaneous injection group, where 50 µl of the solution containing 0.05, 0.1 and 0.2 U of FITC-insulin were injected into abdominal skin. A 29-gauge hypodermic needle and syringe were used and the subcutaneous insertion depth was approximately 2–3 mm; and 3) Control group, where blank MN patches without insulin were applied onto abdominal skin as a negative control. Each group contained five diabetic mice.

To measure BG values continuously, 5 µl of blood sample was collected each time from tail vein laceration at indicated time intervals over a period of 6 h, every 30 min in the preceding 3 h and every 1 h for the next time. One-Touch blood test strips connected with a blood glucose monitor (Lifescan, Milpitas, CA) were used to quantitatively determine the BG values. Additionally, the absorption rate of 0.1 U of FITC-insulin in solid MN and the liquid solution was respectively measured using *In Vivo* Imaging System (IVIS, Caliper life sciences). While being absorbed from the epidermis layer into the deep systemic circulation, the fluorescent signal of the drug delivery area on the back skin of diabetic mice was filtered at excitation wavelength of 445–490 nm and emission wavelength of 515–575 nm, and then observed using a 150 W quartz halogen lamp. The Epi-fluorescence intensity generated from residual FITC-insulin located in the superficial epidermis was recorded every 30 min for the next 4 h, and then transformed into the relative content of FITC-insulin according to the proportional relationship between them. All data were analyzed and expressed as Radiant Efficiency based on IVIS system software.

### Statistical Analysis

All statistical analyses were performed using Origin 8.5 software (Origin Lab Corp, Wellesley Hills, MA, United States). The experimental data were presented with average values, expressed as the mean ± standard deviation (S.D.). The student’s paired *t*-test was used for comparisons between data points. Multiple data sets within groups were analyzed with an ANOVA followed by Dunnett’s post hoc test, when appropriate. For all comparisons, *p* < 0.05 was considered to be statistically significant.

## Results and Discussion

### Fabrication of Insulin-Loaded Microneedles

In this study, a cast processing was used to prepare the drug-loaded dissolving MNs, as shown in Figure 1. The dissolving polymer MNs were fabricated by filling FITC-insulin crystal and PVA/sucrose MN matrix gel into MN cavities with a multi-step vacuum processing technology. Firstly, FITC-insulin solution was filled into the PDMS was mold, and excess insulin solution was collected and reused. After the solution was dried, PVA/sucrose gel was casted on the mold surface, followed by inhaling into the mold under vacuum. Finally, the MN patch was dehumidified and peeled off using an adhesive pad.

**GRAPHICAL ABSTRACT:**
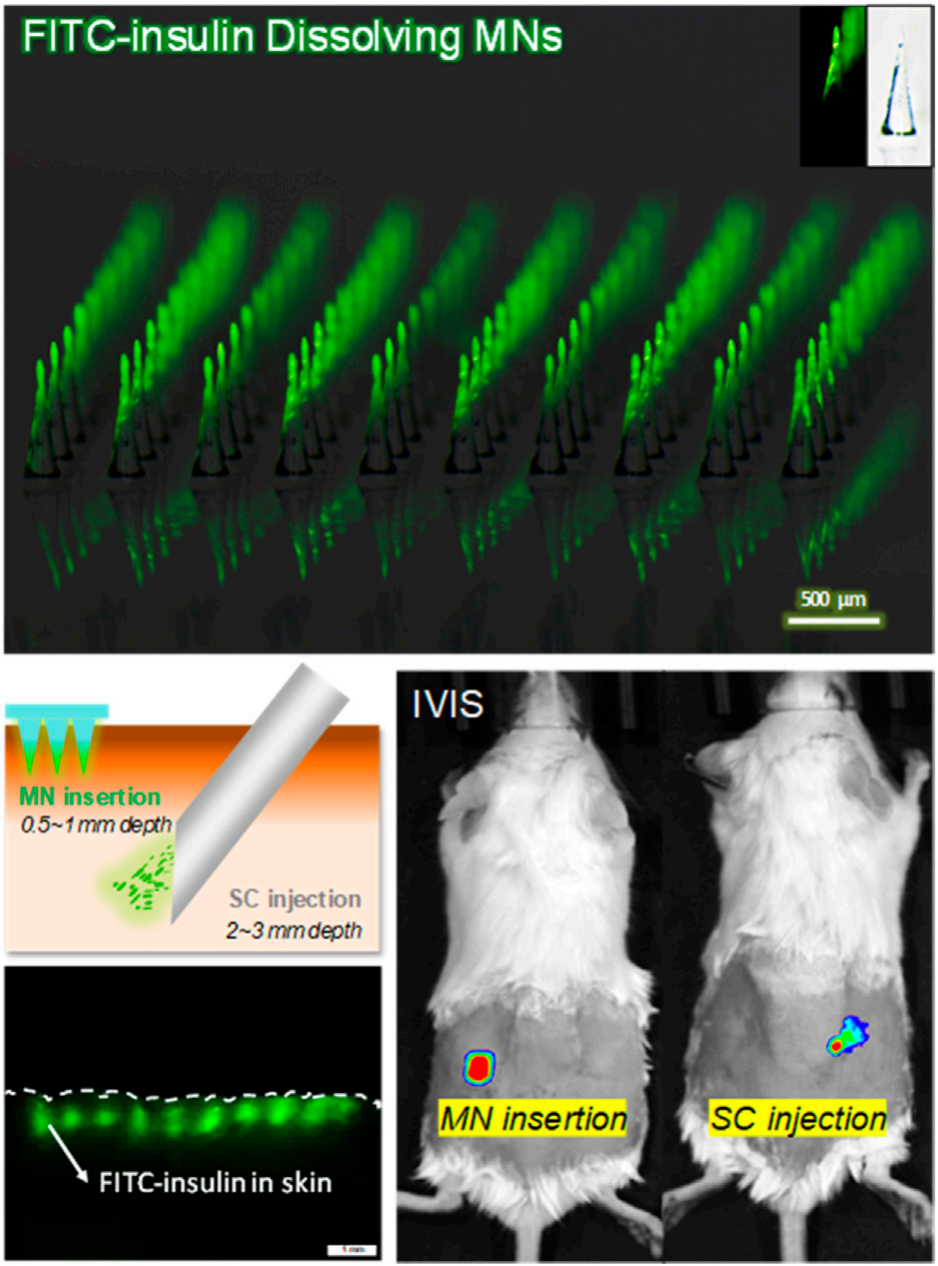


**FIGURE 2:**
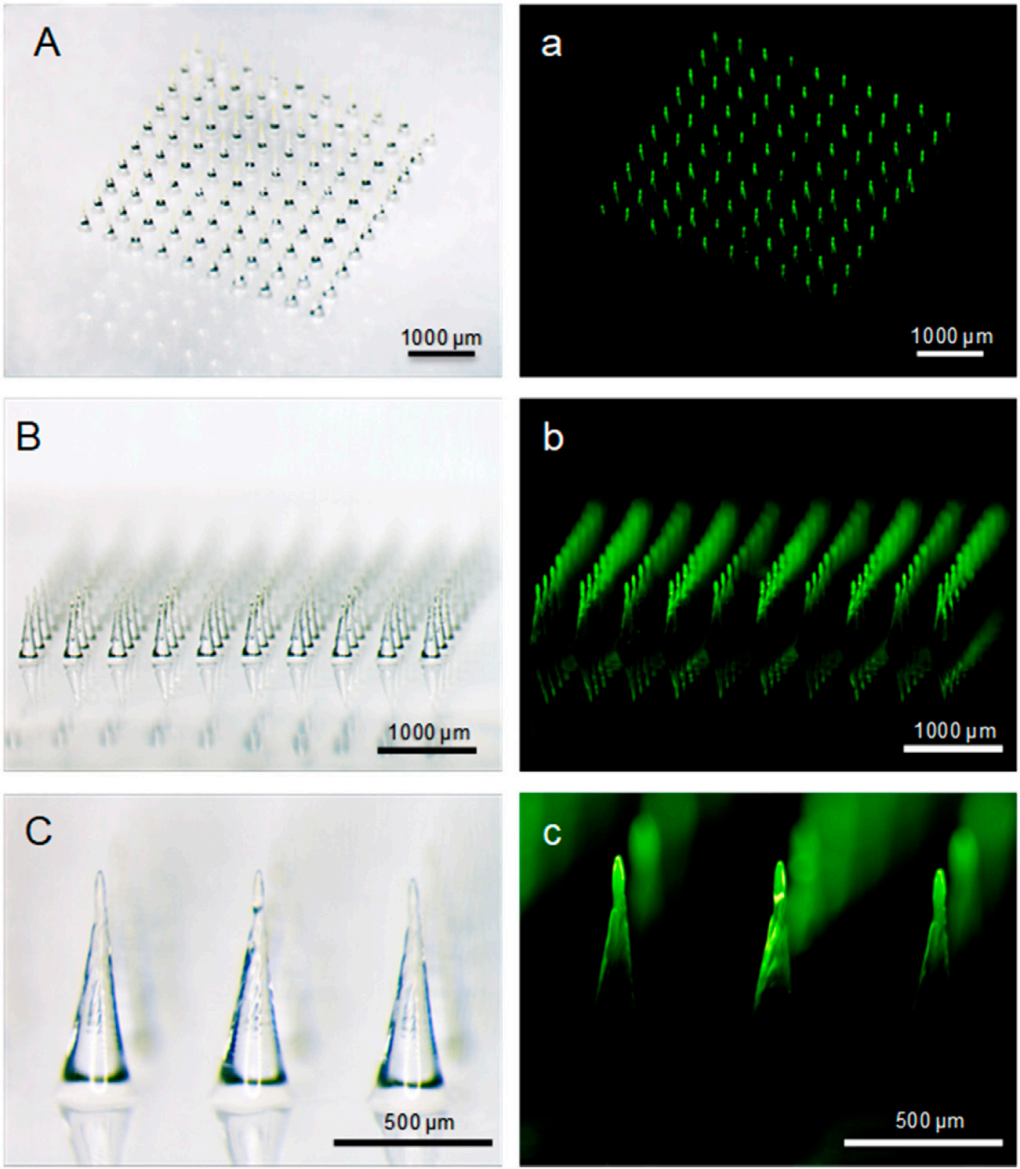
Bright-field **(A−C)** and fluorescent (a, b, c) micrographs of FITC-insulin loaded MN arrays. Scale bar: 500 μm.

Here, PVA and sucrose were used to prepare the backing layer of MN patches, and FITC-labeled insulin was used as an experimental drug. The bright and fluorescent micrographs of MN arrays (Figure 2) indicated that needles integrated into the patch aligned uniformly, and MN arrays showed the same size and geometry as the female MN mold cavities curved with laser device (Wang et al., 2016). Each needle was approximately 750 µm in height, with a diameter of 300 μm at base and 20 μm at the tip, and an interspacing of 500 µm between MN rows. There were 100 needles in the rectangular array with an area of about 0.25 cm^2^. Additionally, FITC-insulin encapsulated in the microneedle patch is mostly located in the needle tips, and the backing layer contained almost no drug with little fluorescent light emission out, not like MN arrays with drug located in the whole needle (Chen et al., 2012; Liu et al., 2012). It was significant to make encapsulated drug distributing mostly in the MN tips because incomplete insertion of dissolving MNs largely limited drug delivery efficiency and led to wastage of valuable medication.

In a previous study, we curved the tapered mold cavities on the flat surface of PDMS membrane using laser-engraving technology (Wang et al., 2016). The geometry of cavities, the size of MN arrays and the interspace of needle tips could be controlled conveniently by adjusting the laser parameters as required. It was beneficial to control the dosage of insulin loaded in the dissolving MNs, which was significant for diabetes treatment. Like many other methods, this fabrication process also avoided organic solvents and heat which was harmful to the bioactivity of insulin. And sucrose in the formulation was capable of protecting insulin from damage as well. In addition, the vacuum processing technology required less energy consumption and more simple equipment to fabricate dissolving MN patches comparing to the air-blowing process or centrifuging methods (Kim et al., 2013; Kim et al., 2016). Considering that, the advantages of PDMS MN mold forming technics and MN patch fabricating process were promising to promote the mass reproduction of dissolving drug-loaded MNs for transdermal delivery of biopharmaceutical macromolecular drugs, especially proteins and vaccines (Lee et al., 2013).

### Mechanical Property of FITC-Insulin Loaded MNs

**FIGURE 3:**
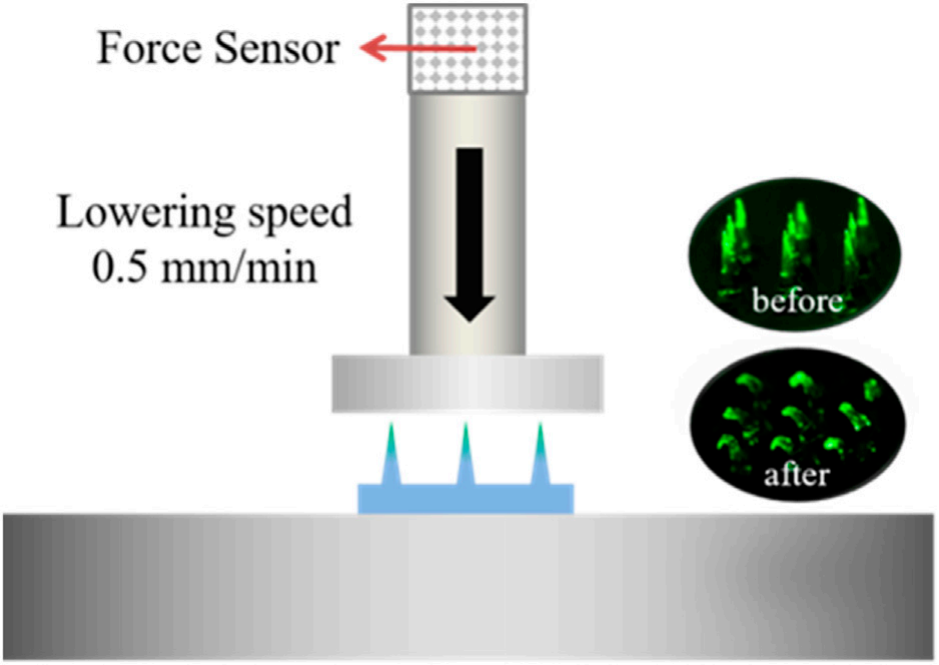
Schematic of testing mechanical property of dissolving MNs. The green fluorescence indicates the FITC-insulin loaded in the MN patches.

**FIGURE 4:**
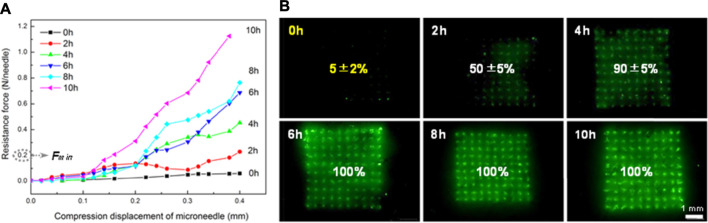
Results of compression test for FITC-labeled MN with different drying time **(A)** and penetration ratio of inserting corresponding FITC-labeled MN arrays into porcine skin successfully **(B)**.

The mechanical property of FITC-insulin loaded MNs was measured using a displacement-force test machine, as shown in Figure 3. The results were collected and shown in Figure 4. Figure 4A showed that MN patches still contained little water just after being peeled off the mold cavities, which resulted in needle tips being too mechanically weak to penetrate the stratum corneum and deliver drugs into the body (0 h curve). To ensure MN arrays pierce into skin successfully, MN patches were dehumidified using a freeze dryer for a specific time to remove the residual moisture. As shown in the force-displacement (F-D) curve (Figure 4A), the greater curve slope represented the stronger mechanical strength of MN arrays. MN patches vacuum freeze-drying for 2 and 4 h exhibited yield points at average failure force of approximately 0.1 N/needle and 0.3 N/needle, respectively. While the general force required to insert into the skin was determined approximately 0.2 N/needle (Park et al., 2005). Additionally, the falling points didn’t appear obviously in F-D curves corresponding to MN patches drying for at least 6 h. These results demonstrated that MN patches drying for at least 6 h obtained sufficient piercing capability.

Furthermore, it was confirmed in accordance with the subsequent MN penetration test *in vitro*. According to the number of fluorescent spots on the skin, complete penetration was observed when drying for 6, 8 and 10 h and incomplete penetration in the group of 0, 2 and 4 h, as shown in Figure 4B. The penetration ratio of inserting corresponding MN arrays into porcine cadaver skin successfully was calculated to be 5 ± 2%, 50 ± 5% and 90 ± 5% respectively. Green spots could be observed only when MN tips penetrated subcutaneous tissue successfully and fluorescent FITC-insulin imported, with residual drug left on the skin surface wiped clean using alcohol pads. These results indicated that MN arrays should be dehumidified in a freeze dryer for at least 6 h before drug administration and then we could obtain the inherent sufficient mechanical strength supported by pure dry PVA and sucrose solid crystal.

### *In vitro* Test for Dissolution Profile of FITC-Insulin Loaded MNs

**FIGURE 5:**
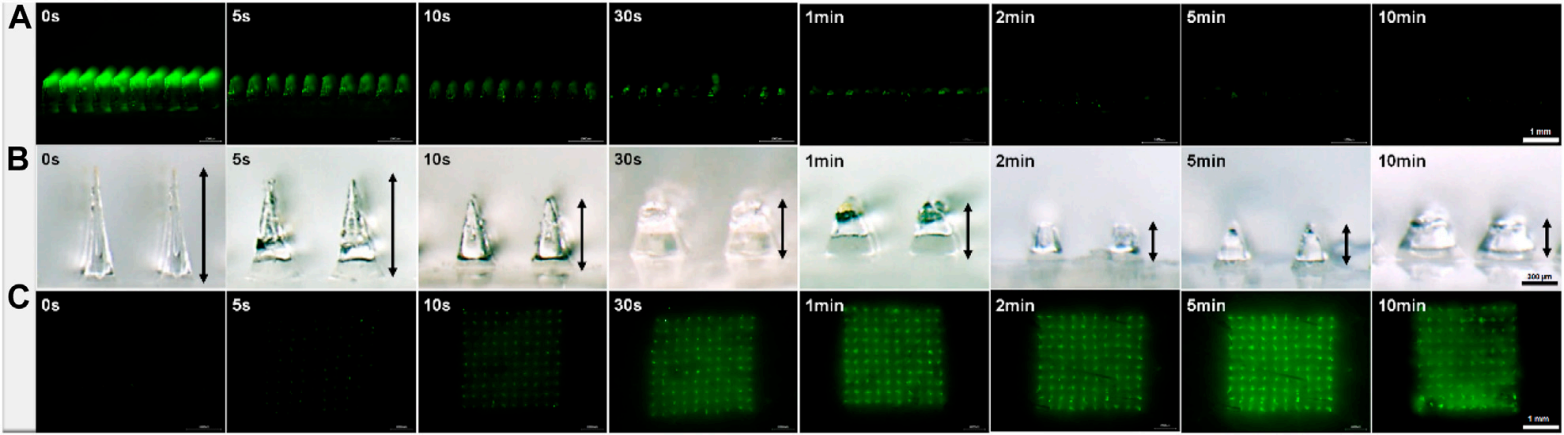
Micrographs of MN arrays after inserting and embedding in subcutaneous tissue with different times **(A, B)** and the images of porcine skin after drug administration *in vitro* with different times **(C)**. Scale bar: 1 mm.

The solubility of MN matrix material affects significantly the administration process of dissolving MNs for transdermal drug delivery. To evaluate the dissolution rate of PVA/sucrose MNs, we inserted prepared FITC-insulin loaded MNs into hairless porcine cadaver skin and kept MN tips embedded in the interstitial fluid for a different time ranging from 5 s to 10 min. All inserted parts of the needles dissolved rapidly in the former 30 s and completely within 2 min (Figures 5A,B). Compared with the HA MNs dissolving completely within 1 h (Liu et al., 2012), or chitosan and PVP MNs within several minutes (Chen et al., 2012; Wang et al., 2015), fast-degrading PVA/sucrose MNs were very efficient and convenient to dissolve and transdermally deliver insulin into subcutaneous tissue. After MN dissolved, encapsulated FITC-insulin was released from the needle tips slowly and PVA/sucrose was absorbed and degraded in the body as other biodegradable dissolving MNs shown (Tsioris et al., 2012; Zhu et al., 2014).

MNs fabricated with PVA (*M*_w_ 6,000 Da) and sucrose in solid-state own sufficient mechanical strength, such as silicon or metal solid MNs, which contributed to piercing tough stratum corneum and forming micro-channels in the skin to deliver drug. Meanwhile, PVA/sucrose mixture was biodegradable and nontoxic to bio-tissue when absorbed into the cardiovascular system. It was significant for dissolving drug-loaded polymer MNs working as pharmaceuticals but not as micro-devices.

### *In vitro* Test of Drug Delivery Efficiency in the Porcine Cadaver Skin

FITC-insulin encapsulated in the MN tips with different doses of approximately 1.65 ± 0.03, 3.34 ± 0.06 and 5.02 ± 0.10 µg/patch were delivered into skin tissue separately, along with conical needles piercing through stratum corneum. To calculate the efficiency of transdermal drug delivery, the average content of FITC-insulin remaining in residual MN substrate and alcohol pads that were used to clean and collect the residual drug on the skin surface was determined as followed, approximately 0.24 ± 0.03, 0.31 ± 0.07, 0.43 ± 0.11 µg/patch respectively, corresponding to the original content in intact MN patches before insertion. Thus, the drug delivery efficiency was 85 ± 2%, 91 ± 2% and 92 ± 3% relatively (Table.1). These results suggested that the overwhelming majority of FITC-insulin was delivered into the skin, which was in accordance with the results of the insertion test *in vitro* (Figure 5). Therefore, it was convenient to control the drug content encapsulated in dissolving MNs accurately by regulating the concentration of drug solution, volume or size of homemade MN mold cavities.

**TABLE 1:** *In vitro* test of FITC-Insulin delivery efficiency in the porcine cadaver skin.

Concentration of drug solution (mg/ml)	Drug content before insertion (µg/patch)	Drug content after insertion (µg/patch)	Drug delivery efficiency (%)
1	1.65 ± 0.03	0.24 ± 0.03	85 ± 2
2	3.34 ± 0.06	0.31 ± 0.07	91 ± 2
3	5.02 ± 0.10	0.43 ± 0.11	92 ± 3

Results are presented as the mean ± S.D. of at least five experiments.

**FIGURE 6:**
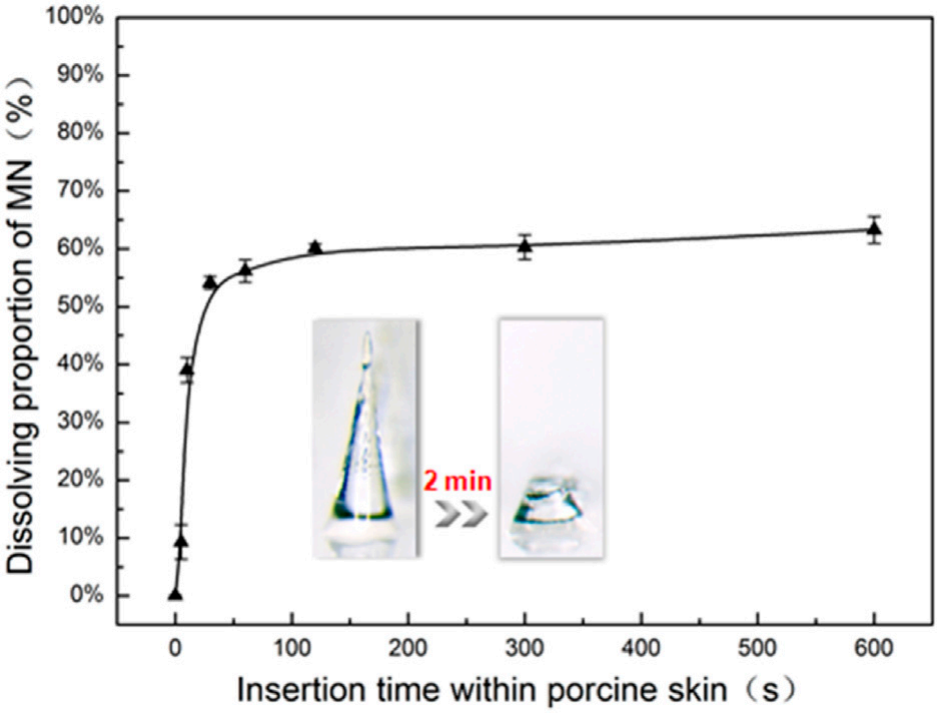
*In vitro* dissolution profile of FITC-insulin loaded MNs inserting into porcine skin with different times (n = 3, mean ± SD).

MNs couldn’t be inserted into the subcutaneous tissue completely because of the deformation and elasticity of the skin in the process of penetration (Chen et al., 2015; Liu et al., 2016). About 40% of the MN length in the base remained in substrates (Figure 6), which means that the FITC-insulin encapsulated in the residual base substrate portion was unavailable to be absorbed into subcutaneous tissue effectively. Moreover, dissolving MNs were mostly applied in the field of transdermal delivery of expensive and unrecoverable drug such as protein and vaccine. So it was important to improve the efficiency of drug delivery and utilization, which was beneficial to control drug dosage precisely and save production costs for the promising industrial application in the future.

As we know, patients with Type I diabetes were dependent on severe insulin therapy (Narayan, 2014). The blood glucose level was sensitive to the variable dosage of therapeutic insulin. Thus, it was essential to treat patients with a certain amount of insulin on their demand in case that overdose insulin causes severe or moderate hypoglycemia or insufficient dosage results in hyperglycemia symptoms (Ly et al., 2013). Compared to traditional subcutaneous injection with the precise hypodermic syringe, the novel way of transdermal drug delivery with MNs had some problems in controlling drug dosage accurately. For instance, coated MNs existed some drawbacks like uncontrollable material deposition on the substrate and weight variations of coating formulations (Gill and Prausnitz, 2007). Moreover, some dissolving MNs with drug distribution in the whole MN led to low drug delivery efficiency. Incomplete insertion of dissolving MNs largely limits drug delivery efficiency and wastage of valuable medication.

### *In vivo* Transdermal Delivery of FITC-Insulin in Diabetic Mice

**FIGURE 7:**
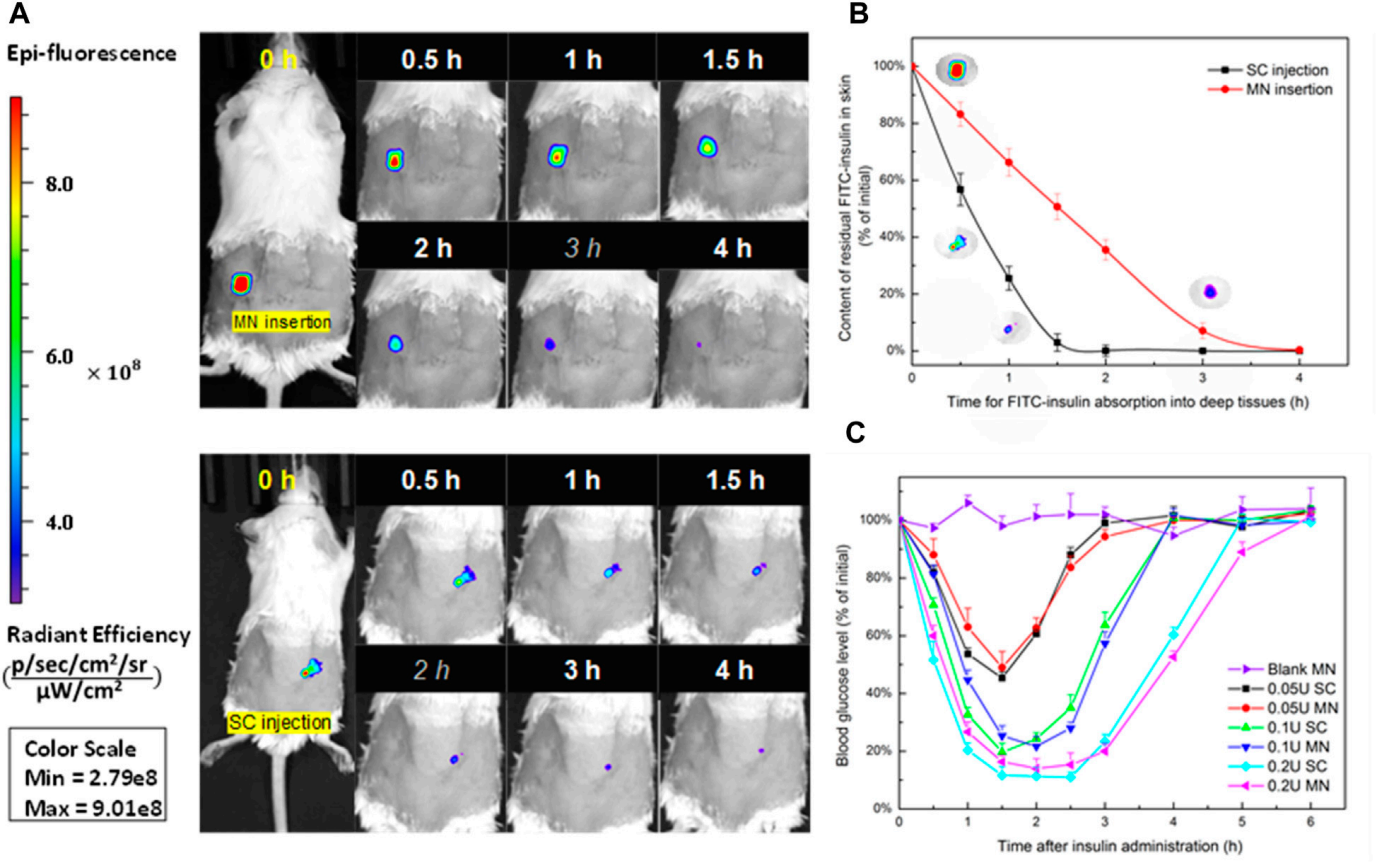
**(A)** Absorption profile of 0.1 U of FITC-insulin delivering into subcutaneous tissue using MN insertion and SC injection respectively. **(B)** Content of FITC-insulin residual in the epidermis layer vs. absorption time after drug administration. **(C)** Blood glucose levels vs. time profiles of diabetic mice after administration of 0.05, 0.1, 0.2 U of FITC-insulin *via* MN insertion and SC injection.

To figure out the absorption rate of FITC-insulin delivered from the subcutaneous epidermis into the deep biological circulatory system, IVIS was used to determine the residual relative content of FITC-insulin on the surface of the epidermis layer. After drug administration, the fluorescent signal intensity generated from original 0.1U of FITC-insulin faded slowly and almost disappeared within 2 h for “SC injection” group and 3 h for “MN insertion” group respectively (Figure 7A, B). Insulin dissolved in solution was absorbed into diabetic mice faster than that encapsulated in dissolving MN patches. This was consistent with the hyperglycemic results that the hyperglycemic effect *via* MN insertion showed a little lag time compared with SC injection of insulin solution (Figure 7C), due to the additional process of solid insulin-loaded MNs dissolving in the interstitial fluid.

To confirm whether FITC-insulin loaded MN patches are effective to reduce the blood glucose level in diabetic mice, we compared two different ways of transdermal insulin delivery *via* MN insertion and SC injection respectively. Figure 7C shown that larger insulin dosage brought better hyperglycemic effect and kept the normal BG level for a longer time in diabetic mice, 0.2 U/patch of insulin made BG level dropt to about 15% (3.5 mmol/L) of the initial value at 1.5 h point and kept the same level for another 1.5 h and then BG level started to increase. In contrast, there were no obvious changes in blood glucose levels in the blank group. MNs pretreated group compared with the control group over the 6 h period. Transdermal delivery of FITC-insulin by inserting prepared MN patches or using a hypodermic syringe to inject drug solution respectively showed no obvious difference in hyperglycemic effect on diabetic mice, which signified that the FITC-insulin loaded PVA/sucrose MNs were as effective as mainstream syringes used by most Type I diabetic patients.

The delicate difference between the absorption rate didn’t affect the pharmacodynamics and pharmacokinetic effect of insulin no matter in solid or liquid state (Chen et al., 2015). And this slightly delayed action of dissolving MNs for insulin delivery provided enlightenment for designing MNs with the function of controlling insulin release slowly into skin tissue, which could be realized by adjusting the molecular weight of PVA matrix to control the solubility and absorption of insulin-loaded MNs, or *via* other methods to control the encapsulated insulin releasing from MNs slowly, like reservoir-based drug delivery systems (Stevenson et al., 2012) or advanced pH-responsive MN patches (Xie et al., 2017). This system may be a promising alternative method for delivering protein drugs, such as insulin, and take the place of traditional hypodermic needles.

## Conclusion

In this study, we introduced a fabrication process of dissolving MNs with FITC-insulin tip-loaded under vacuum condition, using homemade PDMS MN mold carved with laser engraving technology. The geometry and volume of MN mold cavities can be controlled, which is beneficial to control the dosage of drug encapsulated in MNs as required. FITC-insulin MNs fabricated using this method show good capabilities of penetrating the stratum corneum of skin after drying 6 h and delivering insulin into diabetic mice to reduce blood glucose level effectively. The FITC-insulin loaded MNs fabricated in this work have some advantages, such as controllable drug-loaded capacity, fast-dissolving, convenient self-administration, mild and energy-saving fabrication process, etc. showing obvious industrial potential for mass production of drug-loaded MNs in the future.

## Data Availability

The original contributions presented in the study are included in the article/supplementary material, further inquiries can be directed to the corresponding author.
